# Force-Velocity Measurements of a Few Growing Actin Filaments

**DOI:** 10.1371/journal.pbio.1000613

**Published:** 2011-04-26

**Authors:** Coraline Brangbour, Olivia du Roure, Emmanuèle Helfer, Damien Démoulin, Alexis Mazurier, Marc Fermigier, Marie-France Carlier, Jérôme Bibette, Jean Baudry

**Affiliations:** 1 Laboratoire Colloïdes et Matériaux Divisés, UPMC, ESPCI ParisTech, CNRS PECSA UMR 7195, Paris, France; 2 Physique et Mécanique des Milieux Hétérogènes, ESPCI ParisTech, CNRS UMR 7636, UPMC, Université Denis Diderot, Paris, France; 3 Laboratoire d'Enzymologie et Biochimie Structurales, CNRS UPR 3082, Gif-sur-Yvette, France; Stanford University, United States of America

## Abstract

The polymerization of actin in filaments generates forces that play a pivotal role in many cellular processes. We introduce a novel technique to determine the force-velocity relation when a few independent anchored filaments grow between magnetic colloidal particles. When a magnetic field is applied, the colloidal particles assemble into chains under controlled loading or spacing. As the filaments elongate, the beads separate, allowing the force-velocity curve to be precisely measured. In the widely accepted Brownian ratchet model, the transduced force is associated with the slowing down of the on-rate polymerization. Unexpectedly, in our experiments, filaments are shown to grow at the same rate as when they are free in solution. However, as they elongate, filaments are more confined in the interspace between beads. Higher repulsive forces result from this higher confinement, which is associated with a lower entropy. In this mechanism, the production of force is not controlled by the polymerization rate, but is a consequence of the restriction of filaments' orientational fluctuations at their attachment point.

## Abstract 2

The authors propose a new mechanism for actin-based force generation based on results using chains of actin-grafted magnetic colloids.

## Author Summary

Actin self-assembles into filaments, and this produces forces that deform cell membranes in a large number of motile processes. While physical measurements have been performed of the force produced by growth of either a single filament or a large intricate array of filaments organized in an active macroscopic gel, these measurements don't provide a clear picture of how force is produced by the assembly of each filament within a complex structure. The present study explores a situation between these two extremes by measuring the force produced by the assembly of a small number of filaments. We developed a method in which actin filaments grow from the surface of magnetic beads that are aligned by a controlled magnetic field. The distance between beads in a chain-like arrangement increases with time when the force is kept constant. We observe that the growth of actin filaments is not affected by the load, in contrast to the widely accepted “Brownian ratchet model.” Instead, our results suggest that the surface opposite growing filaments imposes restrictions on the rotational fluctuations of a filament at its free hinge anchoring point, inducing a repulsive force. The confinement of filaments increases as they grow, and this in turn increases the repulsive force developed by their growth. This entropy-based mechanism may operate during motile processes when actin networks are loosely organized.

## Introduction

Polymerization and depolymerization of microtubules or actin filaments, in the absence of any molecular motors, generate forces that are relevant to cellular processes, like cell membrane protrusion and propulsion of intracellular pathogens or organelles [1]–[8]. The energy is provided by the difference in chemical potential between the monomers (G-actin) in solution and the subunits incorporated in the filaments. The filament growth should slow and eventually stall as an opposite applied force approaches the thermodynamic limit given by [9]

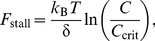
(1)where *k*_B_ *T* is the thermal energy, δ, the elongation distance for the insertion of a new monomer (2.7 nm for actin), *C,* the concentration of monomers in solution, and *C*_crit_, the critical concentration for polymerization.

A good estimate of this limit, at physiological concentration, would be a few piconewtons per filament. Actin filaments in close proximity to a load are thought to elongate through a ratcheting mechanism in which thermal fluctuations, either of the filament end or of the load, allow the stochastic insertion of new monomers in spite of a counteracting force [10],[11]. The theoretical basis of actin-polymerization-generated forces is well developed for a single filament [12] or for a large ensemble of filaments described as a continuous material [13]. However, the individual behavior of filaments in an assembly has only been addressed in numerical simulations and is still under debate [14]–[16], particularly how the macroscopic force is distributed on each filament. Experimental progress has been relatively slow. Convincing experiments have already been reported about the stalling force exerted by single growing actin filaments [17] as well as bundles of filaments [18]. At a much larger scale, the force-velocity profile generated by the growth of a densely branched network comprising thousands of filaments has been measured by several groups [19]–[22]. These experiments give insights into what happens in cells but are too complex to give information on a microscopic mechanism. The force-velocity profile of a controlled, small number of actin filaments has not been measured yet because such experiments require handling short filaments and controlling their organization. The force-velocity profile should be very informative, since its shape is dictated by the microscopic mechanism through which the chemical energy is transduced into force. In this paper we present the force-velocity profile measured for a few actin filaments (typically 10 to 100 filaments) using an original setup. We demonstrate that the force, in our geometry, is due to the entropic restriction of the rotation of the growing filaments at the anchoring points.

## Results

### Force-Velocity Measurements

Here, we have designed an experiment that allows simultaneous measurements of the growth velocity, the loading force, and the elastic response of a few growing filaments. We use 1.1-µm-diameter magnetic colloids that form linear chains when a magnetic field is applied. The distance between the colloids and the magnetic attractive force is accurately monitored through the application of a controlled homogeneous magnetic field. Typically, the force can vary from 0.1 pN to almost 100 pN, while the distance *X* between colloidal surfaces can vary from a few nanometers to several micrometers. The magnetic beads are functionalized with gelsolin, a strong capping protein of actin filament barbed end, at a controlled surface density. The total number of active gelsolins per bead, *N*_GS_, is measured separately. In our experiments we use *N*_GS_ = 4,000 or 10,000, corresponding to mean distances of 33 and 21 nm, respectively, between the gelsolins anchored on the colloids. A detailed characterization of the colloid surface chemistry and a detailed description of our experimental setup are given in Materials and Methods.

**Figure 1:**
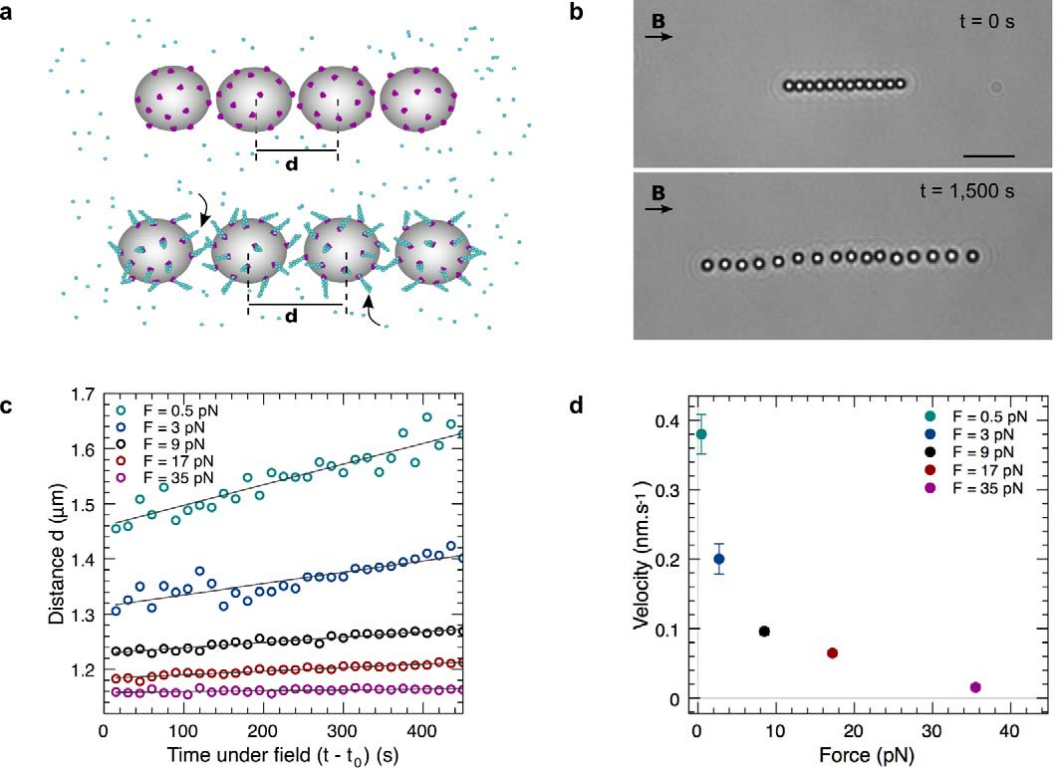
Experimental scheme and representative velocity versus force measurements. (A) Schematics of the experiment: actin polymerization is initiated at the bead surface by gelsolin, and pushes the beads apart. (B) Bright-field images of a colloid chain, aligned under a 5-mT magnetic field, at two different times. Actin filaments are not dense enough to be seen. Scale bar, 5 µm. (C) Evolution of the center-to-center distance *d* with time for different loading forces. The distance increases linearly with time, allowing a direct measurement of the beads relative velocity. (D) Velocity versus loading force profile. Error bars indicate estimated error (standard deviation) from the slope determination in (C). For the largest forces, the error bars are smaller than the symbols. Number of filaments per particle: *N*_GS_ = 10,000.

In the presence of G-actin, gelsolin initiates pointed end growth of filaments at the surface of the beads, as shown schematically in Figure 1A. The radial growth of gelsolin-anchored filaments away from the bead surface causes an increase in the bead-to-bead distance, visualized in video-microscopy images (Figure 1B and Video S1). This qualitative observation clearly indicates that the actin polymerization induces forces larger than a few piconewtons. More precisely, G-actin is first added to the suspension of gelsolin-functionalized magnetic beads at time *t* = 0. Before the application of the magnetic field at *t* = *t*_0_, the filaments grow freely with a pointed end growth rate *v*_0_ up to a length *L*_0_. Figure 1C shows the plot of the beads' center-to-center distance *d* as a function of subsequent time, with constant loading forces ranging from 0.5 pN to 35 pN. *d* increases linearly with time for each force, and therefore the beads' separation velocity *v*_bead_ can be precisely measured as a function of applied magnetic force, *f*. At low forces, this velocity tends to the actin growth velocity in solution derived from kinetics measurements (*v*_0_ = 0.42 nm/s; see Materials and Methods). When the force is increased, the velocity decreases, reaching almost zero at 35 pN (Figure 1D).

### Under Forces the Actin Filaments Grow As If They Were in Solution

**Figure 2:**
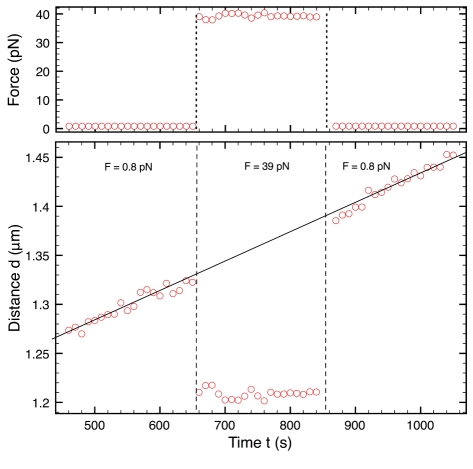
Growth of actin filaments is independent of applied force. The bottom graph shows the evolution of the center-to-center distance *d* as a function of the time *t* for an experiment where the applied force is represented in top graph as a function of *t*. Circles are experimental data. In this experiment, the chain is formed at low force (*f* = 0.8 pN). At time *t* = 650 s the force is increased to a higher value (*f* = 39 pN), and at time *t* = 855 s the force is reduced to the first value. The line is the best linear fit for the points at low forces: *v*_bead_ = 0.302±0.004 nm/s; the intercept is 1,132±3 nm, which is the beads' diameter. *N*_GS_ = 10,000.

In a second experiment, we explored the link between the beads' velocity and the filaments' elongation rate. To address this question, a sequence of low-high-low forces is applied to a chain (Figure 2). During high-force application, the distance *d* between beads is almost constant. However, when the force is suddenly released, the distance increases very rapidly to a value that is higher than the one at the end of the first low-force period (Δd = 63 nm). This result shows that filaments must have grown during the high-force application. Indeed, the observed response is too rapid to be attributed to actin repolymerization. Moreover, the distance versus time before and after application of high force collapse on the same line. This result strongly suggests that the filaments grow as though they were in solution whatever the value of the applied force. In the experiments, the velocity at which the beads separate under force is not directly the velocity at which the actin filaments grow.

### Mechanical Properties of the Grafted Filaments

**Figure 3:**
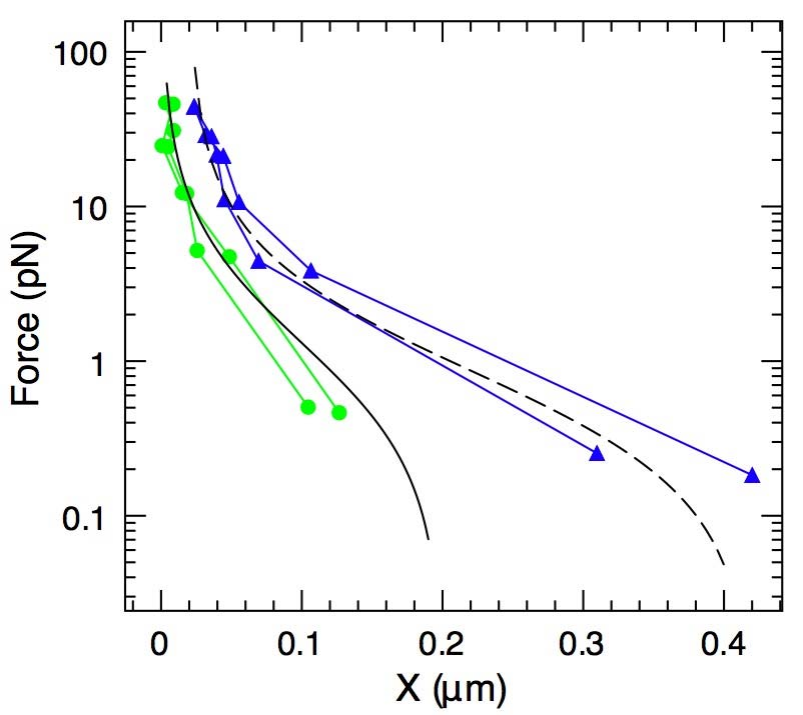
Elastic response of filaments. Instantaneous force-distance profile for different filament initial lengths: applied force *f* as a function of the distance *X* between the surfaces of adjacent beads. Experimental data correspond to a cycle of compression and decompression (*N*_GS_ = 4,000; blue triangles, *L*_0_ = 400 nm and *t*_0_ = 1,020 s; green circles, *L*_0_ = 200 nm and *t*_0_ = 480 s). For clarity, the *L*_0_ = 400 nm data are shifted by 5 nm to the right). Solid and dash lines are predictions of our model with *c* = 0.2±0.1, with *L* = 200 nm and *L* = 400 nm, respectively.

It appears from the previous results that, in our experiments, filaments are not just pushing the beads perpendicular to their surface; their organization in the contact zone is much more complex. Filaments are too short to be directly observed with an optical microscope, so we investigated the detailed mechanical response of our system in an indirect fashion. The instantaneous force-distance profile between colloidal particles within a chain [23],[24] can indeed be obtained using the present setup. At mechanical equilibrium, the net force applied to each bead within the chain is zero, so the attractive magnetic force is strictly balanced by a repulsive force. Hence the repulsive force-distance profile is reconstructed by measuring the interparticle distance *X* between the beads' surfaces at different applied magnetic forces *f*, from 0.1 to 50 pN. We carry out the experiment fast enough to neglect the filament growth. In Figure 3 we present the applied force *f* as a function of *X*. This force-distance profile is measured for two different values of *L*_0_, 200 and 400 nm, over a cycle of compression and decompression. There were three important results of this experiment. First, the mechanical response depends on the initial filaments' lengths. Second, for each length, the two branches of the cycle appear to be equivalent: the force profile is reversible (at low forces the thermal energy induces large fluctuations of the interparticle distance). This indicates that the filaments that build up the repulsive force respond elastically to the load without being damaged by the compressive jump. Finally, as already observed in Figure 2 during switches of the force, the deformation is very high ([*X* − *X*_f = 0_]*/X*_f = 0_ >50%): the elastic modulus of the contact assembly is very small (∼1–10 Pa).

### From Elastic Response to Filament Assembly Organization

**Figure 4:**
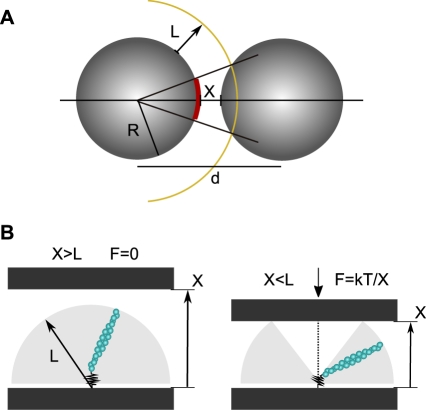
Orientational entropy of the filament at the bead surface. (A) The number of active filaments is estimated from the area of the red surface and the measured filament density. (B) When the distance between black surfaces is large enough (*X*>*L*), the filament explores the whole half sphere shaded on the figure because of thermal fluctuations (left). When *X<L*, the accessible surface Ω decreases (right), leading to a repulsive force.

We will now demonstrate that this soft elasticity is a consequence of the orientational fluctuations of the filaments. The first step is the evaluation of the number of filaments that build up the elastic response. We first evaluated the total number of filaments per bead, *N*_GS_ (Materials and Methods). The number *N* of filaments that can sense the opposite surface is estimated based on a geometrical argument that is illustrated in Figure 4A: a filament of length *L* is counted only if it can touch the opposite bead. We have assumed that the filaments are inter-digitated. This hypothesis is supported by the low concentration of anchoring points and by the fact that the bead velocity at zero force equals the filament's growth velocity in solution (Figure 1): in a tip-to-tip geometry, the distance between beads should increase twice as fast as the filament length. For filaments of length *L*, for a surface-to-surface separation *X*, and for a total number of filaments per particle *N*_GS_, the number, *N*, reads
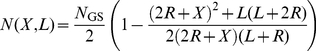
(2)where *R* is the radius of particles. *N* typically ranges from 0 to 250 when *X* is changed from 400 nm down to a few nanometers, for *N*_GS_ = 4,000 and *L* = 400 nm.

We now consider the mechanism that drives the observed soft mechanical behavior (Figure 3). Buckling of filaments is ruled out by their small length (*L*<500 nm). Indeed, a lower estimation of the buckling force *F*_buck_ is the critical Euler force for one filament [12] multiplied by the number of filaments that should buckle 
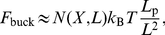
(3)


*L*_p_ being the persistence length (about 8 µm for actin [25]).

*F*_buck_ is about two orders of magnitude larger than the measured force. Since filaments can be truly considered as short rigid rods, we alternatively consider the possibility that elasticity of the system arises from the link between biotin and gelsolin that anchors the filaments to the beads: biotin is bound to gelsolin via a reactive group that contains a flexible spacer of 1.35 nm (see Materials and Methods). This link may act as a free molecular hinge that allows the filaments to be tilted without bending as the opposite surface approaches, as schematized in Figure 4B. Moreover, the free hinge gives rotational degrees of freedom to the filaments.

The elastic response is then hypothesized to be solely due to the entropic restriction imposed by the approaching surface. Indeed, the number of accessible configurations Ω is proportional to the surface area described by the filament tip, and Ω decreases with the confinement: if *X*>*L,* Ω ∝ 2π*L* ^2^, whereas Ω ∝ 2π*LX* if *X<L* (Figure 4B). From the free energy *F* = *−k*_B_ *T* ln Ω, one can compute the repulsive force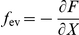. This force is null when there is no confinement, i.e., *X >L*, and is given by *f*_ev_ = *k*_B_ *T*/*X* when *X<L*. We then simply assume that in our case the total force due to *N* filaments will be given by *c N*(*X,L*) *f*_ev_, where *c* is a coefficient that accounts for geometrical effects, i.e., the non-planar surface of the beads. Using the value of *N* given by Equation 2, the forces calculated for the two experimental values of *L*_0_ are compared to the measured ones as shown in Figure 3. The scaling matches the data well, with *c* = 0.2±0.1 as the sole adjustable parameter for both curves.

### Growth of Actin Filaments Develops Entropic Forces

We show here that the force-velocity profile is also governed by the entropic repulsion. If the bead-to-bead distance is kept constant, more filaments are confined as they elongate, and thus the repulsive force increases. In our experiments the force (and not the bead-to-bead distance) is kept constant during filament growth; hence, when the filaments elongate, the distance is modified in a way that keeps the force constant. If the force is exclusively entropy-driven, since *L*(*t*) = *v*_0_ *t*, the bead-to-bead distance *X* can be calculated from an expansion of *N*(*X,L*) at first order in *X*/*R* and *L*/*R*, as follows:
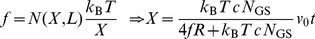
(4)


The assumption that force is entropy-driven yields a linear increase with time of the bead-to-bead distance, in agreement with experimental data (Figures 1C and 2). From Equation 4, we get directly the velocity:
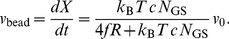
(5)


**Figure 5:**
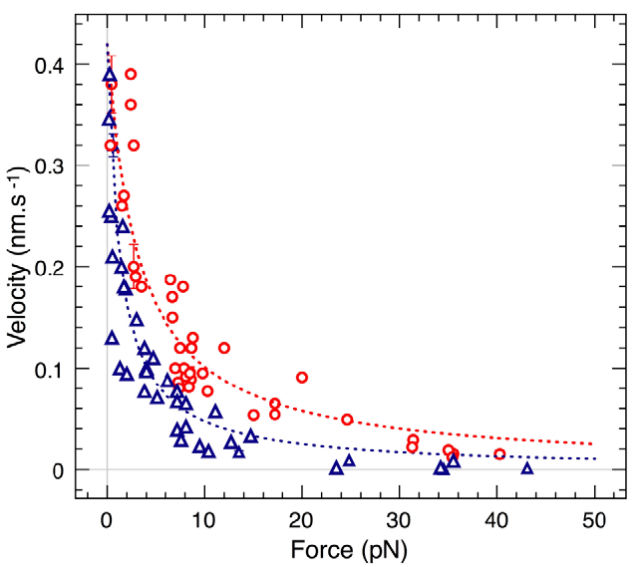
Velocity versus force profiles for different filament densities. Experimental data are discrete open symbols, while the curves are the predictions from our model (red circles, *N*_GS_ = 10,000, 42 measurements; blue triangles, *N*_GS_ = 4,000, 41 measurements).

To further test our model, we performed experiments at different forces and different total numbers of filaments (Figure 5). We also show the comparison of the force-velocity profiles measured and computed from Equation 5 for two values of *N*_GS_, 4,000 and 10,000: both the shape and magnitude match the data very well. The curve fitting gives *c* = 0.18±0.02 for *N*_GS_ = 10,000, and *c* = 0.13±0.02 for *N*_GS_ = 4,000. These *c* values are in good agreement with the *c* value obtained from the mechanical measurements (Figure 3). In addition, this model gives the right limit *v*_0_ = 0.42 nm/s for the velocity at *f* = 0. Finally, according to this model, the velocity never becomes negative even at high forces. Consistent with this, the data actually show that no depolymerization was induced by the applied force.

## Discussion

The force transduction mechanism demonstrated here may be considered as an alternative to the Brownian ratchet model, in a case where large fluctuations in orientation of the growing filament occur as the elongating tip gets close to a surface. In the classical Brownian ratchet model, the growth of the filaments is slowed down by the load that is assumed to be applied to their tip [10], but the organization of the filaments and their perpendicular orientation relative to the surface remain unchanged. In our experiment, the filament growth is not modified by the opposing surface, but filaments orient themselves on average by decreasing their angle to the surface of the magnetic bead with increasing loads. Here the repulsive force pushing the beads away from each other is due to the restriction of rotational freedom around the flexible streptavidin-biotinylated gelsolin linker, which decreases the entropy of the hinged filaments, while they still elongate as in solution. This is analogous to the osmotic pressure, but with orientational degrees of freedom and not translational ones. In addition, this entropy-driven mechanism develops significant forces, typically a few tenths of the theoretical maximum for one filament (see Equation 1).

Does the present mechanism for force production by actin assembly have a physiological relevance in cell motility? Actin arrays that support cell migration are generally oriented with their barbed ends abutting the leading edge, where new filaments are created either by nucleation and processive elongation by formins, or by branching via the WASP/Arp2/3 machinery. Daughter filaments initiated by Arp2/3 branching grow at a 70° angle from the mother filament. Although the branch junction allows some flexibility in orientation of the daughter filament when branched filaments are formed in solution [26], it likely behaves as a more rigid hinge than the streptavidin-gelsolin link in our experiments. The rigidity must also be enhanced by the constraints imposed in the context of the intricate dendritic lamellipodial array [27]. However, some cases exist in which the concepts presented here for force production may apply. Migrating cells actually display a variety of phenotypic morphologies of the lamellipodium [28]. In rapid cell migration of keratocytes, the turnover of a densely branched array feeds fast protrusion associated with a persistent smooth morphology of the leading edge. Filaments keep a constant orientation toward the front, in part because of the interaction of barbed ends with membrane-associated regulators like VASP, which maintain some processive link with growing barbed ends [28],[29]. In the absence of such links, cells migrate with lower directional persistence and the leading edge adopts variable shapes. Within our model, this phenotype may be generated by the greater freedom of reorientation experienced by filaments that would be present in lower number. Similarly, the protrusive activity appears to vary in correlation with variable angles of the filaments to the cell front in the range 15° to 90° [30]. Interestingly, Koestler et al. [30] observed a correlation between the protrusion rate and the filament orientation, similar to our in vitro observations. Hence, depending on barbed end regulation, filament density, and velocity of protrusion, different mechanisms of force production by actin assembly may be at work in migrating cells, and some room may be found for a physiological role of the change in filament orientation in force production.

In conclusion, if the key components at play for cell motility are clearly identified, how their temporal and spatial organizations are regulated in motile processes is still to be unraveled. We believe that our novel experimental approach provides clues to achieve this goal.

## Materials and Methods

### Proteins

Actin was purified from rabbit muscle as previously described [31] and isolated as Ca-ATP-G-actin by Superdex-200 chromatography [32] in G-buffer (5 mM Tris [pH 7.8], 0.1 mM CaCl_2_, 0.2 mM ATP, 1 mM DTT, 0.01 wt% NaN_3_). Ca-actin was converted to Mg-actin by incubation in 0.2 mM MgCl_2_ and 0.25 mM EGTA just before experiments. Actin was pyrenyl labeled as previously described [33].

Recombinant human gelsolin was expressed and purified as previously described [34], and stored in Tris buffer at −80°C (20 mM Tris [pH 7.5], 1 mM EGTA, 0.15 M NaCl_2_, 0.01 wt% NaN_3_). Protein was first dialyzed in a PBS buffer with 1 mM EGTA and 0.01 wt% NaN_3_ (pH 7.5), then biotinylated with sulfo-NHS-biotin (EZ-Link Sulfo-NHS-Biotin Reagents, spacer arm length 1.35 nm; Thermo Scientific) during 45 min at room temperature. Biotinylated gelsolin was used immediately after preparation. To determine the molar ratio of biotin to protein, the HABA method was used (Pierce Biotin Quantitation Kit; Thermo Scientific) and gave 15 biotins per gelsolin.

### Actin Kinetics and Thermodynamical Parameters

The critical concentration *C*_crit_ and the growth rate *k*_on_ at the pointed end in our salt conditions were both derived from pyrene fluorescence assays [35]. Fluorescence polymerization measurements were performed using 2 µM monomeric actin (10% pyrenyl-labeled) in the polymerization buffer (5 mM Tris, 40 mM KCl, 0.6 mM MgCl_2_, 0.2 mM CaCl_2_, 0.2 mM ATP, 1 mM DTT, 0.5 wt% F-127, 0.01 wt% NaN_3_ [pH 7.8]). *C*_crit_ = 0.7 µM was given by the equilibrium concentration of serially diluted F-actin samples in the presence of gelsolin. *k*_on_ was assayed from kinetic measurements using 1:2 gelsolin-actin (GA_2_) complexes at different concentrations. Fitted exponential curves give *k*_on_ = 0.12 µM^−1^·s^−1^. From our working concentration of monomeric actin (*C* = 2 µM), we computed the polymerization velocity from the pointed end in solution, *v*_0_ = *k*_on_δ(*C − C*_crit_) = 0.42 nm/s, and stalling force for a single filament, *F*_stall_ = (*k*_B_ *T/*δ)ln(*C/C*_crit_) = 1.6 pN.

### Sample Preparation

Streptavidin-coated superparamagnetic particles of 1.135 µm in diameter (5 µl) (Dynabeads MyOne Streptavidin, 10^6^ streptavidins per particle; Dynal-Invitrogen; size measured by dynamic light scattering method) were washed five times in Tris buffer containing the pluronic surfactant F-127 (5 mM Tris [pH 7.5], 0.5 wt% F-127, 0.01 wt% NaN_3_). Then, they were incubated with 1 µM freshly biotinylated gelsolin for 10 min for the maximal density. Next, 0.5 mM biotin is added to the sample to block streptavidin free sites. After 5 min, the grafted particles were washed five times with 5 mM Tris (pH 7.8), 0.2 mM CaCl_2_, 0.5 mM biotin, 0.2 mM ATP, 1 mM DTT, 0.5 wt% F-127, 0.01 wt% NaN_3_, and stored in the same buffer. A fraction of the grafted particles were used to determine the grafting density (see below). Next, 0.01 wt% grafted particles were mixed with 2 µM monomeric actin in the polymerization buffer. The obtained solution was rapidly transferred into a capillary tube (Vitrocom) that was sealed at both ends and attached to a slide with liquid wax. Force-velocity measurements and gelsolin biotinylation were performed on the same day. One sample was used for one point in the velocity-force diagram, and up to ten samples could be made with one gelsolin preparation.

### Bead Characterization

The total number of active gelsolins per bead, *N*_GS_, was obtained from pyrene-actin fluorescence assay. Actin polymerization was induced by adding 0.01 wt% gelsolin-coated beads to 2µM G-actin in the polymerization buffer. The number of growing filaments, and therefore *N*_GS_, was computed from kinetic measurements using the measured association rate at the pointed end: *k*_on_ = 0.12 µM^−1^·s^−1^. We used *N*_GS_ of 4,000 or 10,000 in our experiments, corresponding to a mean distance between gelsolins of 33 nm and 21 nm, respectively. Before force measurements, filaments were allowed to grow freely on the beads until *t*_0_. The initial length of the filaments *L*_0_ was directly deduced from this time, *L*_0_ = *v*_0_ *t*_0_, where *v*_0_ is the pointed end growth velocity in solution.

### Force and Distance Measurements

The chains of typically ten beads were imaged using a Nikon TE-2000 inverted optical microscope. Two electromagnetic coils, mounted onto a motorized stage (ECO-STEP; Märzhäuser), generated a magnetic field from 0 to 100 mT. Images were collected through a 100× oil immersion objective (N.A. 1.25) using a digital camera (ORCA-ER; Hamamatsu). The bead positions were obtained with a particle tracking algorithm using NIS-Elements Nikon software [36]. The mean interparticle distance was then calculated by averaging the distance between particles within the chain, and the magnetic force was calculated from the magnetic field and the mean distance *d* according to [24]. Electrostatic repulsive forces between beads under our conditions (40 mM KCl) are negligible as compared to our measured forces. For instantaneous force-distance profiles, a cycle of compression-decompression takes about 2 min to complete, allowing about 50 nm of growth for the filaments. For velocity-force profiles, the mean distance was measured every 15 s, and the magnetic field was adjusted in order to keep the magnetic force constant.

### Data Analysis

The experiments were performed with several purifications of actin, two different batches of magnetic beads, and two different purifications of gelsolin, without noticeable difference. Velocity-force curves are shifted to lower velocity if the actin preparation is too old (more than 3 wk).

For force-velocity measurements (Figure 5), the main variability is coming from the grafting procedure, inducing changes in the gelsolin density. Measurements are more reproducible within the same batch of grafted beads (six points from one preparation at 7.3±0.1 pN, *v*_bead_ = 0.076±0.011 nm/s; ten points from two preparations, *f* = 7.2±0.5 pN, *v*_bead_ = 0.126±0.04 nm/s), but the whole profile cannot be obtained with a single preparation.

For force-distance measurements (Figure 3), the main source of error is the polydispersity of the beads (1% in size), since we are using typically ten beads in a chain. From one chain to another, the force profiles are identical, but can be shifted on the *x*-axis by typically 10 nm. We indeed removed the mean bead diameter to obtain *X* from the center-to-center measurements. This 10-nm shift in *X* impacts the *c* value obtained by fitting the data; an error of 0.1 for *c* is a conservative estimation.

## Supporting Information

Video S1 Magnetic colloids move apart as a consequence of actin polymerization. Video of the magnetic chain during actin polymerization.
(9.52 MB AVI)
